# Vision-Related Quality of Life and Self-Rated Satisfaction Outcomes of Rhegmatogenous Retinal Detachment Surgery: Three-Year Prospective Study

**DOI:** 10.1371/journal.pone.0028597

**Published:** 2011-12-05

**Authors:** Haidong Zou, Xi Zhang, Xun Xu, Haiyun Liu, Lin Bai, Xian Xu

**Affiliations:** Department of Ophthalmology, Shanghai First People's Hospital, Shanghai Jiaotong University, Shanghai, People's Republic of China; Washington University School of Medicine, United States of America

## Abstract

**Background:**

Subjective functional outcomes measurements, such as vision health-related quality of life (VRQoL) and self-rated satisfaction measures can provide helpful multidimensional vision health information that is more comprehensive than traditional objective measures, such as best corrected visual acuity (BCVA). The purpose of this study is to demonstrate 3-year longitudinal postoperative VRQoL and self-rated satisfaction changes after rhegmatogenous retinal detachment (RRD) surgery.

**Methodology/Principal Findings:**

A prospective case series report was conducted in 92 RRD patients who underwent surgery during January 2004 through December 2006. Preoperative, 3-month, 1-year and 3-year postoperative patient VRQoL and self-rated satisfaction were assessed by face-to-face interviews. The importance of objective variables for predicting three dependent variables: CLVQOL composite scores change, 3-year postoperative CVLQOL composite score and self-rated satisfaction degree scores were calculated by stepwise multivariate linear or logistic regression analysis methods.

**Results:**

The total CLVQOL composite scores change ranged between -48 and 90 (mean±standard deviation: 19.48±31.34), including positive changes in 62 patients. The self-rated satisfaction degree scores ultimately improved in 86 patients as compared with preoperative degrees. Statistically significant increases occurred only in the composite scores of subscale mobility and self-rated satisfaction degrees in the first 3 months, while the composite scores of the remaining subscales, and the total CLVQOL, BCVA in the RRD eye and weighted average BCVA, increased steadily throughout the first postoperative year. A better 3-year postoperative weighted average BCVA was associated with all of the 3 dependent outcome variables.

**Conclusions:**

VRQoL of RRD patients improved substantially after surgery and they were satisfied with their postoperative vision. The BCVA, VRQoL and self-rated satisfactory degree scores recovered in different patterns, and supplemented each other in the RRD surgery outcomes evaluated. Surgeons are advised to pay closer attention to binocular vision in RRD patients, and make efforts to explain the results of surgery.

## Introduction

Although the anatomic success rate of rhegmatogenous retinal detachment (RRD) surgery has improved over recent decades, limitations in visual function remain primarily because of permanent functional damage once the macula becomes detached (macula-off RRD). [1] Best corrected visual acuity (BCVA) is the most common indicator of RRD surgical outcomes; however, ophthalmologists nowadays recognize that patients are more interested in how interventions affect their wellbeing than in improvements in biomedical indicators. Thus, vision health-related quality of life (VRQoL), including physical functioning and social functioning have received increasing attention as useful supplemental outcome measures. Until recently, an understanding of VRQoL results after RRD surgery was based on retrospective investigations, [2]–[7] with the drawbacks of inevitable recall bias. Furthermore, the postoperative investigation period in most studies was not longer than 12.7 months. [3]–[7] Since visual acuity after retinal reattachment may continue to improve over a long period of time (43.5 months or 5 years), [8], [9] the long-term VRQoL changes after RRD surgery inspire much debate.

In a previous study, we investigated the VRQoL scores in 163 Chinese subjects with newly diagnosed RRD. [10] We continued to follow these RRD patients. The purpose of the present prospective study is to demonstrate the 3-year longitudinal postoperative VRQoL and self-rated satisfaction changes in these patients.

## Methods

### Participants

Inclusion criteria of the present study were: Chinese patients who took part in the previous study [10] and later underwent RRD surgery in our department; 140 eligible patients were then invited to postoperative investigative visits. The postoperative investigation period started from the primary RRD surgery, or the last RRD surgery (including silicone oil removal in retinal reattached eyes) if multiple procedures were involved in the same eye, and ended after 3 years. During the 3-year investigation period, 48 of 140 patients were excluded because 10 patients underwent additional cataract surgery and 38 patients failed to participate in a timely manner. The final 92 enrolled participants did not differ from the 48 non-participants in the major socio-demographic or clinical characteristics (independent *t* sample test, Mantel-Haenszel chi-square test or one-way ANOVA test, all *P*>0.10), as shown in Table 1.

**Table 1:** Preoperative socieodemographic, clinical, CLVQOL and self-rated satisfaction data, and primary surgery data of 140 eligible RRD patients*.

	Participants	Non-participants
No. of patients	92	48
Age years [Mean(SD)]	49.7 (14.4)	53.3 (11.9)
Male [No. (%)]	50 (54.4)	28 (58.3)
Education time > 10 years [No. (%)]	59 (64.1)	28 (58.3)
Duration of symptoms		
< = 1 week	20 (21.7)	10 (20.8)
> 1 week and < 1 month	47 (51.1)	22 (45.8)
> = 1 month	25 (27.2)	16 (33.3)
Weighed average logMAR BCVA [Mean(SD)]	0.51 (0.50)	0.42 (0.24)
Proliferative vitreoretinopathy grade		
A, B [No. (%)]	82 (89.1)	44 (91.7)
C, D [No. (%)]	10 (10.9)	4 (8.3)
More than 2 quadrants detached [No. (%)]	43 (46.7)	21 (43.8)
Macula detached (macula-off) [No. (%)]	60 (65.2)	30 (62.5)
Degrees of patient-rated satisfaction		
Scale 1 very dissatisfied [No. (%)]	72 (78.3)	32 (66.7)
Scale 2 moderate dissatified [No. (%)]	12 (13.0)	8 (16.7)
Scale 3 moderate satisfied [No. (%)]	8 (8.7)	8 (16.7)
CLVQOL composite score [median (range)]	96 (30–125)	92 (43–122)
RRD primary surgery methods		
Scleral buckle [No. (%)]	53 (57.6)	25 (52.2)
Vitrectomy [No. (%)]	39 (42.4)	23 (47.9)
Retinal reattachment after primary RRD surgery [No. (%)]	76 (82.6)	37 (77.1)

*CLVQOL: Chinese-version Low Vision Quality of Life Questionnaire, RRD: rhegmatogenous retinal detachment, BCVA: best corrected visual acuity, SD: standard deviation.

### Procedures or Investigations

The collected socio-demographic and clinical data included patient age, gender, education time after kindergarten, duration of RRD symptoms to RRD surgery (or, if no symptoms were noted, time since diagnosis), proliferative vitreoretinopathy grade, [11] extent of retinal detachment in quadrants, macula-on or macula-off, RRD surgery types and procedures, intraoperative and postoperative complications. The type of surgery was dichotomized into two categories: scleral buckle, and vitrectomy with or without scleral buckle in any of the procedures. The preoperative, 3-month, 1-year and 3-year postoperative BCVA data were also collected, including Snellen BCVA, logMAR BCVA converted from Snellen BCVA fractions [12] in the RRD eye, and weighted average logMAR BCVA. The latter represented a summary score of BCVA encompassing visual information from both eyes, with the better eye given a weight of 0.75 and the worse eye a weight of 0.25. [4]

We used the Chinese-version Low Vision Quality of Life Questionnaire(CVLQOL) to assess the multidimensional VRQoL of the patients. The closed-ended 25 visual-functioning items in the CLVQOL were grouped into four subscales: (1) general vision and lighting (GL, 7 items assessing fatigue adaption, light/dark adaption, glare disability, and visual search for street signs, television, or moving objects ), (2) mobility (M, 5 items assessing depth or distance judgment, outdoor activities including seeing curbs and crossing roads, and overall movement vision function), (3) psychological adjustment (PA, 4 items: unhappy with the situation in life, restricted in performing certain tasks or visiting friends, and how well the eye condition was explained), and (4) reading, fine work and activities of daily living (RFA, 9 items assessing vision function of reading large prints, newspapers, labels, letters and time from the clock or watch, writing, using tools and self-care activities). The items were all graded on an ordinal score between 5 (no problems due to vision) and 1 (great difficulties due to vision). These items can also be scored as no longer possible due to vision (attributed a grade of 0), or as not relevant to patients in their daily lives (attributed the average score of their total responses to avoid bias in the results of those who had fewer relevant items relevant than others). The CLVQOL composite scores ranged from 125 (representing best vision function) to 0 (representing binocular NLP). [13] Global assessment questions were addressed to the patients regarding their degree of satisfaction with their vision on a scale of 1 to 4, where 1  =  very dissatisfied, 2 = moderately dissatisfied, 3 = moderately satisfied and 4  =  very satisfied. A skilled interviewer (H.Z), who did not participate in clinical observations, conducted face-to-face interviews of the preoperative, 3-month, 1-year and 3-year postoperative CLVQOL and self-rated satisfaction questions, and ensured the consistency of the methodology. He also collected additional comments on the explanation of surgery and its expected results as adequate or not adequate, at the end of investigation period.

### Ethics

All of the enrolled participants gave written informed consent for participation in this study. This study was conducted according to the tenets of the Declaration of Helsinki, and was approved by the Institutional Review Board at the Shanghai First People's Hospital, affiliated Shanghai Jiaotong University.

### Statistical methods

SPSS V10.0 Statistical package (Chicago, IL) was used for database setup and the analyses. Participants were compared with non-participants using the independent samples *t* test for continual data and the Mantel-Haenszel chi-square test or one-way ANOVA test for the categorical data. The same tests were also performed to compare age, gender, education time, duration of RRD symptoms, proliferative vitreoretinopathy grade, extent of retinal detachment in quadrants, macula-on or macula-off, preoperative logMAR BCVA in the RRD eye and weighted average logMAR BCVA, intraoperative and postoperative complications between the scleral buckle group and the vitrectomy group. In our previous study, the reliability and repeatability of the CLVQOL were examined among 100 participants with low vision, and the Cronbach's *α* coefficients and split-half coefficients for the four scales and total CLVQOL questionnaire were calculated as between 0.75 and 0.97. [13] However, there were only 27 patients with various retinal diseases (including 4 RRD patients) in the 100 participants, and the sample size is not enough for reliabity estimation of CLVQOL in people with RRD. Therefore, using pre- and postoperative CLVQOL item scores, the Cronbach *α* coefficients and split-half coefficients were calculated in this study to ensure the internal consistency reliability of CLVQOL measurements in RRD patients. We considered a Cronbach's *α* coefficient or a split-half coefficient over 0.7 to represent a reliable scale. The consecutive differences between preoperative, 3-month, 1-year and 3-year postoperative BCVA, CLVQOL scores, and self-rated satisfactory degrees were estimated respectively. BCVA results were presented as mean (standard deviation) logMAR fractions and compared with Paired samples *t* test. CLVQOL score results, which did not follow normal distribution, were presented as median (range) scores and compared with the nonparametric Wilcoxon signed rank test. The patient numbers with certain satisfaction degrees were presented and compared with the nonparametric Wilcoxon signed rank test. All tests were two-tailed, and *P*< 0.05 was considered statistically significant.

The importance of many objective variables for predicting three dependent variables: (1) change in CLVQOL composite scores calculated as 3-year postoperative composite scores minus preoperative composite scores, (2) 3-year postoperative CVLQOL composite score and (3) self-rated satisfaction degree scores were calculated with different regression analysis methods. The change in CLVQOL composite scores, representing long-term VRQoL changes due to RRD surgery, should have followed Gaussian distribution and been modeled with stepwise multivariate linear regression analysis methods. Three-year postoperative CVLQOL composite scores and self-rated satisfaction degree scores representing final outcomes, which did not follow Gaussian distribution, were modeled with logistic regression analysis methods. To fit the model, CLVQOL, the composite score, was dichotomized to high (scores of 107 or more) and low (score of 106 or less), and the self-rated satisfaction was dichotomized to satisfied (degree 3 or 4) and dissatisfied (degree 1 or 2). The choice of a cut-off of 107 was based on our former observation of mean CLVQOL composite score in 100 randomly selected normal vision people.^13^ The independent variables included age (< = 60 or >60 years), gender, education time (< = 10 or >10 years), macular detachment (on or off), duration of symptoms (< = 1 week, 1 week to 1 month or >1month), extent of detachment in quadrants (< = 2 or >2 quadrants), proliferative vitreoretinopathy grade (A and B, or C and D), type of RRD surgery, surgical procedures (only one or more), surgery complications (yes or none), preoperative CLVQOL composite score, preoperative and 3-year postoperative BCVA in the RRD eye and weighed average BCVA. In the linear regression model, the logMAR BCVA fractions were used for calculation. In the logistic regression model, the logMAR BCVA fractions were stratified into 5 groups: 0 to 0.1, 0.17 to 0.4, 0.48 to 0.7, 1 to 1.3 and larger than 1.3 (equivalent to Snellen fractions 20/20 to 20/25, 20/30 to 20/50, 20/60 to 20/100, 20/200 to 20/400, and worse than 20/400, respectively), because logistic regression demands an interval level or categorical independent variable. Independent variables were allowed in the linear regression model with *P*-entry value as 0.05 and *P*-exit value as 0.10, and in logistic regression model if *P* < 0.05.

## Results

Twelve of the 92 participants were diagnosed with bilateral RRD. In the fellow eyes of 4 patients, early stage macula-on RRD without significant symptoms or vision impairment was observed, and retinal re-detachment was later achieved after one scleral buckle surgery. Long-term undiagnosed RRD with severely impaired vision (Snellen visual fraction <0.05) was observed in the fellow eyes in 8 patients, who abandoned fellow eye RRD surgery due to personal reasons. Since the visual acuities hardly changed in the fellow eyes during the 3-year investigation period, we only present herein the clinical data of the first diagnosed RRD eye, and have reason to suppose VRQoL and self-rated satisfaction changes were due to the first diagnosed RRD eye surgery. All the RRD surgeries were conducted during January 2004 through December 2006. In the 92 participants, the retinas reattached in 76 eyes within the primary surgical procedure (82.6%), in 85 eyes (92.4%) within two procedures, and in 89 eyes (96.7%) within three procedures. Three patients abandoned surgeries for retinal re-detachment due to personal reasons. Mild postoperative complications were found in 39 eyes (42.4%), including temporary hypertension in 20 eyes, mild cataract in 12 eyes, flat translucent epimacular membrane in 4 eyes, and temporary corneal edema in 3 eyes. Statistically significant relationship was observed between the two surgery groups and different proliferative vitreoretinopathy grade, extent of retinal detachment in quadrants, macula-on or macula-off, preoperative logMAR BCVA in the RRD eye and weighted average logMAR BCVA, intraoperative and postoperative complications (independent *t* sample test, Mantel-Haenszel chi-square test or one-way ANOVA test, all *P*<0.05).

In the 92 participants, the Cronbach α coefficients and the split-half coefficients of the preoperative, 3-month, 1-year and 3-year postoperative CLVQOLs were 0.94 and 0.91, 0.93 and 0.88, 0.93 and 0.87, 0.93 and 0.90, respectively. None of the BCVAs, scores of subscales and total CLVQOL, or self-rated satisfaction degree scores changed significantly between the 1-year and 3-year postoperative observation times (Paired samples *t* test or Wilcoxon signed rank test, all *P* >0.05). In the first postoperative year, these indexes changed in different patterns. The changes of BCVA in the RRD eye, the weighed average BCVA, the scores of subscales GL, PA and RFA, and total CLVQOL composite scores increased steadily throughout the first year (Paired samples *t* test or Wilcoxon signed rank test, between preoperative and 3-month postoperative time, 3-month and 1-year postoperative time, both *P*<0.05). Scores of subscale M, and self-rated satisfaction degree scores increased only in the first 3 months (Paired samples *t* test or Wilcoxon signed rank test, between preoperative and 3-month postoperative time, *P*<0.05, and between 3-month and 1-year postoperative time, *P>*0.05) (Table 2). The change in total CLVQOL composite scores ranged between -48 and 90 (mean±standard deviation: 19.48±31.34), including positive changes in 62 patients and none or negative changes in 30 patients. The 3 patients who had largest negative changes (−48, −37, −36) included 2 patients who abandoned surgeries for retinal re-detachment, and 1 patient with postoperative epimacular membrane development. They were all macular-on RRD patients and underwent vitrectomy surgery. The preoperative BCVA of the RRD eyes all decreased sharply at the end of 3-year postoperative time, from 0.4 to 0.05, 0.3 to 0.01 and 0.4 to 0.2 (with metamorphopsia), respectively. As a comparison, the 3 patients who had largest positive changes (90, 88, 87) were all macular-off RRD patients and underwent successful vitrectomy surgery, without any intraoperative or postoperative complications. The preoperative BCVA of the RRD eyes increased sharply at the end of 3-year postoperative time, from 0.03 to 0.5, 0.05 to 0.5, and 0.08 to 0.6, respectively. By stepwise linear regression analysis, a higher CLVQOL composite scores change was associated with a lower preoperative CLVQOL score, a better 3-year postoperative weighted average BCVA, worse preoperative weighted average BCVA, and better 3-year postoperative BCVA in the RRD eye (cumulative adjusted R square were 0.32, 0.37, 0.43 and 0.46, all *P*<0.05). By logistic regression analysis, a higher 3-year postfoperative CLVQOL composite score was associated with a better 3-year postoperative weighted average BCVA and macula-on preoperatively (regression coeffiecients were 3.82 and 1.75, both *P*<0.05). The subgroup comparison of CLVQOL composite scores between macula-on and macula-off patients are shown in Table 3.

**Table 2:** Preoperative and postoperative logMAR BCVA, CLVQOL scores and self-rated satisfaction degrees in 92 RRD patients*.

	Preoperative	3-month Postoperative	1-year Postoperative	3-year Postoperative
logMAR BCVA [mean (SD)]				
in the RRD eye	1.13±0.72	0.70±0.51	0.55±0.36	0.53±0.40
weighted average	0.51±0.50	0.38±0.44	0.32±0.36	0.32±0.35
CLVQOL composite scores [median (range)]				
General vision and lighting	27 (10–35)	32 (13–35)	32 (17–35)	32 (18–35)
Mobility	21 (6–25)	23 (12–25)	23 (12–25)	23 (12–25)
Psychological adjustment	10.5 (4–20)	17 (7–20)	18 (12–20)	17.5 (10–20)
Reading, fine work and activities of daily living	37 (1–45)	37 (9–45)	40 (19–45)	40 (21–45)
total	96 (30–125)	109 (50–125)	111.5 (68–125)	111 (67–125)
Self-rated satisfaction [No.]				
very dissatisfied	72	5	0	2
moderate dissatisfied	12	8	4	2
moderate satisfied	8	17	30	30
very satisfied	0	62	58	58

*CLVQOL: Chinese-version Low Vision Quality of Life Questionnaire, RRD: rhegmatogenous retinal detachment, BCVA: best corrected visual acuity, SD: standard deviation.

**Table 3:** Preoperative and postoperative total CLVQOL composite scores [median (range)] in 32 macula-on and 60 macula-off RRD patients*.

	Preoperative	3-month Postoperative	1-year Postoperative	3-year Postoperative
macula-on patients	101 (48–125)	121 (98–125)	122 (107–125)	121.5 (103–125)
macula-off patients	92 (30–122)	104.5 (50–122)	109 (68–122)	107.5 (67–121)

*CLVQOL: Chinese-version Low Vision Quality of Life Questionnaire, RRD: rhegmatogenous retinal detachment.

The self-rated satisfaction degree scores finally improved in 86 patients as compared with preoperative degrees. Sixty-one of the the 86 patients (70.9%) experienced a positive total CLVQOL composite scores change, and the remaining 25 patients (29.1%) did not experience such a change, but all agreed that the explanation of their RRD surgery and its expected results were adequate. By logistic regression analysis, higher 3-year self-rated satisfaction degree scores were associated with better 3-year postoperative weighted average BCVA and better 3-year postoperative BCVA in the RRD eye (regression coefficients were 0.53 and 0.24, both *P*<0.05).

## Discussion

RRD is a rapid-onset ocular disorder, and it is always difficult to determine the preoperative VRQoL status, because patients may not experience their functional status for more than a few hours or days before surgical intervention. [4], [6] In Chinese RRD patients, both their access to medical facilities capable of conducting RRD surgery and their knowledge about this blinding disease are limited, which leads to a long duration of RRD symptoms before surgery, hence facilitating preoperative VRQoL and self-rated satisfaction assessments. Based on our MEDLINE literature search, ours seems to be the first prospective report clearly documenting VRQoL changes due to RRD surgery.

CLVQOL is one of the few well-developed Chinese Language VRQoL measurement instruments for Chinese people with low vision. It was originally translated from the English-language Low Vision Quality of Life Questionnaire (LVQOL), [14] and then adapted into Chinese culture. The CLVQOL has been proven to satisfy conventional psychometric criteria, be able to discriminate visually healthy populations from those with low vision, and has the potential to identify benefits of low vision rehabilitation. [13] The high Cronbach α coefficients and the split-half coefficients calculated in the present study further indicated that this measure has acceptable reliability for group-level comparisons in RRD patients. These reliability scores were similar to what we formerly calculated among 100 participants with low vision. [13] Although there are still some controversies on how to correlate the VRQoL change with certain item score alterations in the questionnaire, summarizing the ordinal items by averaging to confer the functional improvements after RRD surgery was proven to be valid in previous studies. [2], [6], [7] According to the statistically significant increase in the CLVQOL scores, we identified a substantial VRQoL recovery in the first postoperative year and different recovery patterns in multi-dimensional vision functions. The vision functions relating to subscale mobility (M) seem to recover only in the first 3 months after RRD surgery. The common symptom of RRD is a "shadow" or "curtain" (scotoma) spreading from the peripheral visual field, and stereopsis was not impaired extensively in a majority of early stage RRD subjects. This is why the vision functions mainly relevant to stereopsis, such as seeing curbs and judging the depth or distance, decreased mildly in RRD patients compared with those with normal vision. [10] Therefore, it appears logical that these visual functions rapidly recovered after successful RRD surgery and have limited potential to increase during the subsequent investigation period. The other vision functions and the mental health of the RRD patients were harmed more severely, [10] and tended to recover steadily throughout the first postoperative year. The endpoint of RRD surgery follow-up periods is usually set as 3-month or 6 months, because most patients are thought to have stable visual acuity 3 to 6 months after surgery. [1] However, according to the VRQoL and BCVA data in the present study, we recommended that surgeons follow RRD patients for at least one year, and explain to the patients before RRD surgery that improvement of vision function may require this period of time.

The increase in postoperative self-rated satisfaction degree score did not parallel the positive VRQoL change or BCVA increase. As Ranta and associates discussed in their report, this indicated that both types of patient related outcomes (subjective and objective) supplement each other and are useful adjuncts when evaluating the success of vitreoretinal surgery. [2] Approximately 30% of the final satisfaction was ascribed to proper explanation of surgical results, which emphasizes the importance of appropriate interaction between the patient and surgeon. [3], [4]

The results of linear regression analysis provided evidence for our intuitive hypothesis: the better the final BCVA and the lower the preoperative BCVA, the more likely patients were to report improved functional status. The postoperative weighted average BCVA was found independently related to all of the 3 outcome indicators, which suggests that the functional outcomes measures are also highly dependent on the fellow eye, and the surgeons pay more attention to the BCVA of the fellow eye and to binocular vision in RRD patients. [2], [5] It is commonly recognized that visual acuity recovery after macula-on detachment is dramatically better then in macula-off detachments, [1] and the VRQoL recovery showed a similar difference (Table 3). Based on regression analysis results, the macula-on or -off status was considered to have an influence on the finial visual function outcomes, but not on VRQoL change, since the probability of functional improvement was limited in macula-on patients with better preoperative functional status.

The patients always had severely impaired BCVA and a detached macula at primary vitrectomy surgery, and faced high risks of intraoperative and postoperative complications. However, the surgery type was not proven to be associated with CLVQOL composite scores change, 3-year postoperative CLVQOL composite score, or 3-year self-rated satisfaction degree scores after regression analyses. Therefore, we consider that most of the patients only care the final visual recovery after RRD surgery, but not the complexity of the surgery.

Some shortcoming of our study should be mentioned. First, because a majority of the enrolled participants complained of restrictions in reading preoperatively, [10] the CLVQOL was administered by an interview method at that time; therefore, all the postoperative CLVQOL followed the same method of administration to ensure consistency. The interview method itself may lead to potential bias. For example, patients tend to assign a higher grade in the face of doctors. For this reason, we used the interviewer who did not participate in any clinical treatment of the patients, in order to control the bias. Secondly, the preoperative administration of a questionnaire may lead to inevitable familiarity with some items in some patients at early postoperative times, and these patients may overestimate the 3-month CLVQOL item scores. Nevertheless, this recall bias would be reduced to a large extent until the 1-year investigation time. Third, most of the enrolled Chinese patients suffered a long duration of RRD symptoms before surgery, which resulted in the high proportion of macula-off RRD. Thus, the present study may not reflect the overall experience of persons undergoing RRD surgical procedures. In future work, more subjects with a different preoperative status may help to shed further light on the VRQoL and satisfaction in patients undergoing surgery for RRD.
